# From survival of irradiated mice to modern molecular insights: a seventy-year journey in radiobiology at the institute of biophysics, Czech academy of sciences

**DOI:** 10.1007/s00249-025-01765-9

**Published:** 2025-06-13

**Authors:** Jiří Toufar, Lucie Toufarová, Iva Falková, Alena Bačíková, Martin Falk

**Affiliations:** Department of Cell Biology and Radiobiology, Institute of Biophysics of the Czech Academy of Sciences, Kralovopolska 135, Brno, Czech Republic

**Keywords:** Institute of biophysics of the Czech academy of sciences, Radiobiological research, Biological effects of ionizing radiation, DNA damage and repair, Chromatin architecture at micro- and nano-scale, Photon radiation, Densely ionizing (high-LET) particle radiation, Microscopy, Single molecule localization microscopy (SMLM)

## Abstract

This paper has been prepared to commemorate the 70th anniversary of the Institute of Biophysics of the Czech Academy of Sciences (IBP CAS), which has a long-standing tradition in researching the biological effects of ionizing radiation (IR). Radiobiology has recently gained renewed importance due to several compelling factors. The demand for a better understanding of the biological effects of both low and high doses of various types of ionizing radiation, along with improved radiation protection, is increasing—particularly in the context of critical ongoing human activities such as medical diagnostics, radiotherapy, and the operation of nuclear power plants. This demand also extends to newly emerging scenarios, including the development of hadron and FLASH radiotherapy, as well as mixed radiation field exposures related to planned manned missions to Mars. Unfortunately, there is also an urgent need to address the heightened risk of nuclear materials and weapons misuse by terrorists or even rogue states. Additionally, nuclear energy is currently the only viable alternative that can provide efficient, sustainable, and ecological coverage for the dramatically increasing current and future energy demands. Understanding the risks of IR exposure necessitates exploring how different types of IR interact with living organisms at the most fundamental level of complexity, specifically at the level of molecules and their complexes. The rising interest in radiobiology is, therefore, also driven by new experimental opportunities that enable research at previously unimaginable levels of detail and complexity. In this manuscript, we will address the important questions in radiobiology, focusing specifically on the mechanisms of radiation-induced DNA damage and repair within the context of chromatin architecture. We will emphasize the differing effects of photon and high-LET particle radiation on chromatin and DNA. Both forms of IR are encountered on Earth but are particularly significant in space.

## Introduction: the increasing importance of radiobiology and radiation protection in contemporary contexts

Since the discovery of X-rays on November 8, 1895, scientists and the general public alike have been captivated by the biological effects of this mysterious radiation, and subsequently, ionizing radiation of various types. Initially celebrated for their applications in medical diagnostics, X-rays soon revealed harmful effects on tissues and organs, which paved the way for the development of cancer radiotherapy (Baatout 2023). Concurrently, it became evident that ionizing radiation possesses a dual nature: while it can effectively eradicate tumors, it may also inadvertently stimulate their growth (Baatout 2023).

**Fig. 1:**
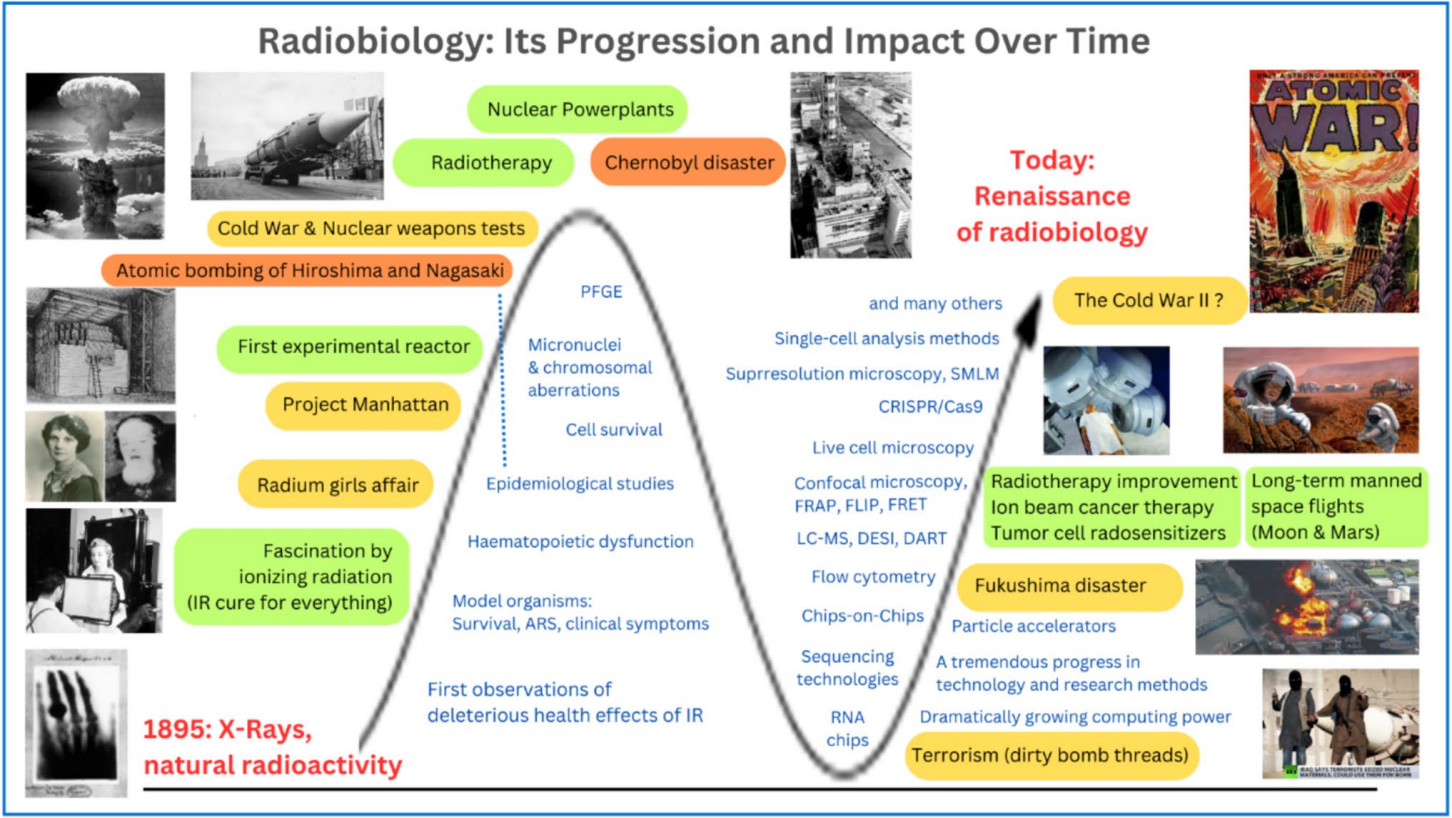
Radiobiology—its progression and impact over time. Radiobiology is once again becoming a topic of significant attention for several reasons: 1. The search for new, more effective, and safer cancer radiotherapy techniques. 2. Issues related to medical imaging (multiple CT scans, CT-PET, etc.). 3. The increased risk of (tactical) nuclear weapons usage by criminal states. 4. Exposure of crews to ionizing radiation as a critical concern in the context of planned manned missions to Mars. 5. The small yet non-negligible possibility of accidents at nuclear power plants with severe consequences, as well as at other facilities emitting IR; development of new nuclear reactors for ecological energy production. 6. A relatively heightened risk of terrorist attacks using dirty bombs. 7. Radon exposure, and 8. Other critical areas of human activity. The significant events shaping radiobiology research are highlighted in black text. The sinusoidal curve represents the fluctuating public interest in ionizing radiation research, correlating with these key events. Notable research methods and devices that propelled advancements in understanding radiation and its biological effects are depicted, though not strictly aligned with their historical occurrence. *ARS* acute radiation syndrome, *PFGE* pulse-field gel electrophoresis, *LC-MS* liquid chromatography–mass spectrometry, *DESI* desorption electrospray ionization coupled with MS, *DART* direct analysis in real time coupled with MS, *FRAP* fluorescence recovery after photobleaching, *FLIP* fluorescence loss in photobleaching, *FRET* fluorescence resonance energy transfer, *SMLM* single molecule localization microscopy.

In the ensuing period (Fig. 1), interest in radiobiology has varied depending on numerous influencing factors (Wojcik and Harms-Ringdahl 2019), but research into the biological effects of ionizing radiation (IR) and strategies for mitigating these effects has remained a central focus at the Institute of Biophysics of the Czech Academy of Sciences (IBP CAS). In honor of the 70th anniversary of this institute, the present article seeks to initiate a comprehensive discussion on the current challenges in radiobiology. We will focus specifically on the effects of IR on chromatin at micro- and nanoscale levels. This includes examining the chromatin’s response to irradiation and exploring how these cellular effects translate into broader health implications.

In recent decades, particularly in Europe, radiation research has been complicated by unwarranted radiophobia. The industrial applications of IR have unfortunately been accompanied by several significant disasters (Fig. 1), the most notable being the Chernobyl nuclear disaster, which remains the most infamous catastrophe globally (Baverstock and Williams 2006), followed by the Fukushima disaster (Funabashi and Kitazawa 2012; Thielen 2012). Although other accidents, such as those in the chemical industry, can rival even Chernobyl in terms of direct casualty counts and health consequences for survivors (Grimston 2002; Dhara and Dhara 2002; Broughton 2005), the long-term contamination of vast areas with radionuclides leads to uninhabitability, thus giving radiation events a unique character (Grimston 2002). In addition, the imperceptibility of ionizing radiation to our senses, coupled with the stochastic nature of the long-term risks associated with even low levels of exposure, lends it an elusive and even mysterious quality. This characteristic significantly contributes to the psychological impact of radiation disasters and radiophobia.

Paradoxically, radiophobia can lead to even more tragic consequences than the radiation accident itself. For example, several European countries experienced a significant increase in induced pregnancy terminations following the Chernobyl disaster (Perucchi and Domenighetti 1990; Knudsen 1991; Spinelli and Osborn 1991), despite the negligible direct health risks associated with radioactive fallout in Europe (Drozdovitch et al. 2007; Yeager et al. 2021). Similarly, no lives were lost due to radiation exposure following the Fukushima Daiichi nuclear accident, both among the general public and the disaster responders. However, some people died during evacuation (see below) or due to untreated naturally occurring diseases, as many individuals began to reject radiation-based diagnostic procedures (Tamaki and Shishido 2011). The victims of radiation disasters have also experienced enduring stress and psychological issues for the reasons mentioned above, which have, in some cases, manifested as real physical illnesses. Additionally, survivors of the Chernobyl disaster and the tragic atomic bombings of Hiroshima and Nagasaki—referred to as Hibakusha—faced social stigma and exclusion due to public misunderstanding about the mechanisms of radiation damage, leading to the misconception that irradiated individuals remained dangerous (Tanigawa et al. 2012; Hasegawa et al. 2016).

**Fig. 2:**
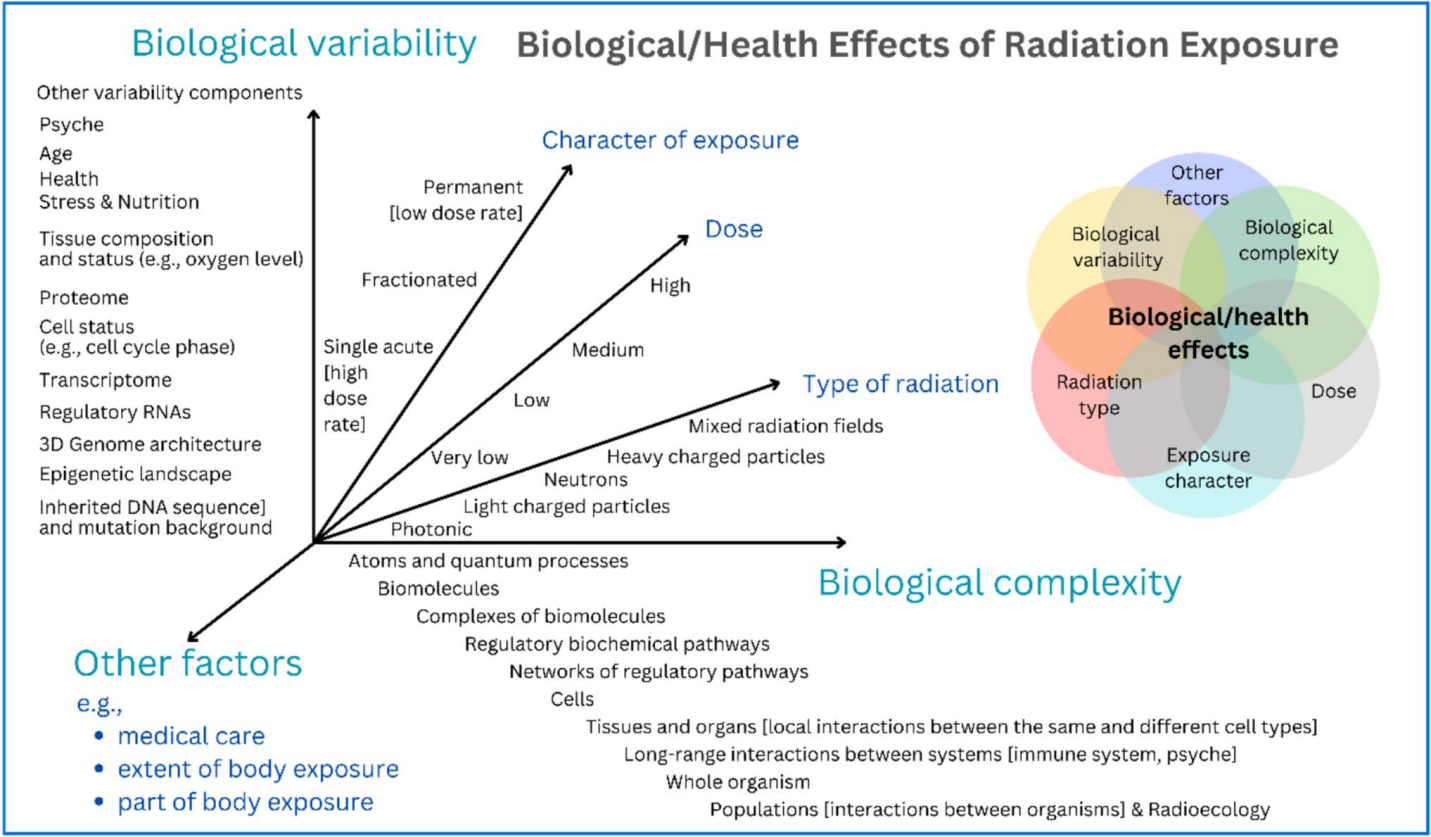
The complex and variable nature of biological effects induced by ionizing radiation is illustrated in this multidimensional plot, which highlights the key factors that influence radiation-mediated biological outcomes. It is important to note that the processes leading to biological and health effects begin with physical interactions occurring at approximately 10^−18^ s and may culminate decades later, potentially manifesting as cancer development

The psychological factors can also significantly impact the decision-making processes in managing radiation disasters. As an example, mentioned above, some elderly individuals and patients in intensive care did not survive the evacuation of more than 100,000 people, which was ordered as a preventive measure after the Fukushima Daiichi disaster (Tanigawa et al. 2012; Hasegawa et al. 2016). All these paradoxical victims of radiation accidents highlights the complexity of ionizing radiation’s health effects on humans, encompassing not only direct radiation damage but also various types of bystander effects (Morgan 2003; Mancuso et al. 2012; Morgan et al. 2016; Daguenet et al. 2020) and psychological aspects (Bromet et al. 2011). Moreover, this complexity is compounded by individual differences at each level of biological organization, including DNA, cells, and tissues (Fig. 2) (Falk et al. 2014a, b). A better understanding of both the complexity of radiation effects and the multi-level variability of biological responses to irradiation is essential for preventing radiophobia while planning and developing effective countermeasures for radiation protection and nuclear accidents (Sihver and Yasuda 2018; Nagashima and Yasuda 2020, 2021).

Currently, there is a prominent resurgence of interest in the study of the biological effects of ionizing radiation (IR), driven by several important factors (Fig. 1). First, the incidence of various cancer types is increasing in the developed world (Kanavos 2006; Sung et al. 2019; Gupta et al. 2020; Cronin et al. 2022; Hughes et al. 2024) (though the reasons for this are still debated, including considerations such as improved diagnostics and population aging (Jemal et al. 2011)), and some of these cancers remain particularly challenging to treat, with radiotherapy still being one of the most widely used and best therapeutic modalities. Second, as medical imaging develops, repeated X-ray exposures, CT scans (including preventive whole-body CT examinations with very disputable benefits), PET and CT-PET examinations become an everyday issue in modern medicine; this dramatically increased the contribution from medical diagnostics to the average annual effective dose per person (Akram and Chowdhury 2025). Third, the deteriorating geopolitical climate poses substantial risks related to the misuse of nuclear materials or even weapons by terrorists or rogue states (Huang 2024). Fourth, undertaking planned manned missions to the Moon and Mars will expose astronauts to mixed radiation fields, which represent one of the primary obstacles associated with these flights (Committee on the Evaluation of Radiation Shielding for Space Exploration, Aeronautics and Space Engineering Board, Physical Sciences 2008). Fifth, nuclear power is currently being developed as the only viable alternative to replace fossil fuels in an effective and sustainable manner. Thus, the need for radiobiological research emerges from contexts that exemplify both the pinnacle of human achievement and, conversely, a profound disregard for humanity. This paradox elucidates why radiobiology has often been imbued with an underlying sense of apprehension since the initial excitement (Fig. 1).

Among the mentioned exposure scenarios reinforcing the importance of radiobiological research, the inhalation of radon must not be overlooked, as it represents the most significant source of natural radiation exposure to humans (Grzywa-Celińska et al. 2020)). Radon inhalation is notable for several reasons: (a) it leads to accumulation of radon products in lungs followed by emission of alpha particles, which are particularly dangerous in cases of internal contamination (Falk and Falkova 2020; Baatout 2023), (b) it is the most common inducer of lung cancer, especially in combination with smoking (Sethi TK et al. 2012; Yoon et al. 2016; Lorenzo-González et al. 2019; Urrutia-Pereira1 et al. 2023), (c) it is the only natural source of IR that individuals can significantly control, and d) it provides valuable data for large populations exposed to varying doses of IR over extended periods. Conversely, there is emerging but fundamentally statistically limitted evidence suggesting that ionizing radiation (IR) may confer certain beneficial effects on human health (Donaubauer et al. 2024). Such positive effects have been observed in regions characterized by elevated background radiation levels (Hosoda et al. 2021; David et al. 2021), as well as following therapeutic radon spa treatments (Maier et al. 2020; Donaubauer et al. 2024). The studies indicate that exposure to low doses of radiation may stimulate biological responses that can enhance immune function and tissue repair (Mavragani et al. 2016; Lumniczky et al. 2021). Furthermore, the research highlights the potential for low-level radiation to be associated with a decreased risk of certain diseases, including cancer (Hosoda et al. 2021; David et al. 2021). These findings contribute to a growing body of literature investigating the dual nature of IR’s impact on health.

At the Institute of Biophysics of CAS (https://www.ibp.cz/en/research/departments/department-of-cell-biology-and-radiobiology/info-about-the-department), early top-secret research efforts focused on identifying new radioprotective agents capable of reducing the risk of acute radiation syndrome (ARS), relevant to both the civilian population and especially, in the context of the Cold War, to soldiers on the battlefield. Given the technological capabilities of that era, the effects of the studied substances were assessed at the levels of whole (model) organisms, specifically focusing on survival rates and hematopoietic function. The modern studies in this direction can be represented by (Hofer et al. 2016, 2017b, 2017a).

To fully understand the risks and benefits associated with radiation exposure, it is essential to elucidate the mechanisms by which ionizing radiation (IR) impacts biological systems, starting from the molecular level. This includes the damage inflicted on biomolecules, particularly DNA, and their associated complexes, such as chromatin and biological membranes. Recent technological advancements have enabled the investigation of DNA damage and repair mechanisms following exposure to various types of IR at the micro- and even nanoscale levels (Falk et al. 2007, 2008b, 2014a, 2014b; Depes et al. 2018; Hausmann et al. 2018, 2021a, 2022; Jezkova et al. 2018; Weidner et al. 2023). This area of research has now become a central focus of radiation studies at the Institute of Biophysics, Czech Academy of Sciences. This article will, therefore, concentrate on the initial DNA damage and repair processes triggered by IR within the context of chromatin architecture and the subsequent events that lead to radiation effects at higher levels of biological organization. These effects may ultimately manifest as either deterministic or stochastic health outcomes (Falk and Falkova 2020; Baatout 2023). Moreover, we will discuss why different types of ionizing radiation exhibit varying biological effectiveness regarding their impact on human health and their ability to eradicate tumor cells in the context of radiotherapy (Barendsen 1994; Joiner 2009).

The following discussion is primarily based on our experiments conducted at radiation doses typically exceeding 1 Gy. Since a dose of approximately 0.7 Gy is considered the threshold for the development of acute radiation syndrome (ARS) (Havránková 2020; Baatout 2023), it can be stated that the results discussed are particularly relevant for higher and high doses of ionizing radiation. However, it is essential to emphasize that health risks associated with exposure to low and very low radiation doses pose a critical concern in radiation biology and radiation protection because of two principal reasons:

1. Low-dose exposure is an everyday reality for the general population, exemplified by increasing exposure from medical diagnostics (such as CT, PET, and CT-PET scans) and radon. The growing popularity of preventive full-body CT scans, particularly in the United States (Dong et al. 2025), cannot be overlooked.
2. Our understanding of the mechanisms underlying low-dose exposures remains insufficient. The nanoscale changes in chromatin architecture discussed in this review (Weidner et al. 2023; Falk et al. 2025a, b) may represent a newly identified mechanism contributing to phenomena such as low-dose-associated hypersensitivity and genomic instability (Smith et al. 2003; Kochanova et al. 2023), or, according to other studies, hyposensitivity, more complex dose-effect relationships, and even hormesis (Falk et al. 2014a).

Given that, at a dose of 1 Gy, a single cell is typically traversed by only one particle, it can be inferred that the results obtained—particularly regarding particle radiation—also apply to very low doses, where irradiated cells are affected by a single particle while the proportion of unexposed cells increases as the dose decreases.

## Diverse types of ionizing radiation: unraveling the connection between physical characteristics and biological effects

First, let us introduce the types of ionizing radiation and examine the mechanisms by which they induce biological effects.

Early empirical observations demonstrated that, for a given dose, there are significant differences in biological effects among various types of radiation. The subsequent studies have established that these differences arise from the distinct physical properties of each radiation type and, consequently, the mechanisms by which they interact with and damage DNA. Alongside traditional photon-based radiotherapy (Šlampa 2021), proton therapy is becoming increasingly prevalent (Durante 2019; Michaelidesová et al. 2020), while the use of accelerated carbon ions is also being explored, albeit less frequently due to their technical challenges and cost implications (Ebner and Kamada 2016; Dokic et al. 2016; Durante et al. 2017). For equivalent therapeutic doses, gamma rays and protons produce markedly different therapeutic outcomes compared to accelerated carbon ions, primarily due to the fundamentally different nature of the chromatin (DNA) damage they induce (Falk et al. 2014b; Durante et al. 2017; Jezkova et al. 2018; Hausmann et al. 2021b).

Furthermore, various exposure scenarios present unique mixed radiation fields, each posing distinct risks to human health. For instance, (a) during a nuclear explosion, an individual would be exposed to an intense combination of gamma radiation and neutrons released as initial radiation, followed by diverse types of radiation (alpha, beta, and gamma) emitted from radioactive decay products, resulting in radioactive fallout (residual nuclear radiation) (Solomon; et al. 1986). In addition, the released neutrons can activate surrounding materials (Lee et al. 2022), creating additional radiation sources. (b) In the context of space exploration, particularly during interplanetary missions, crews operating beyond Earth’s protective magnetic field will encounter a complex mixture of energetic gamma photons, protons, alpha particles, and various accelerated ions (Takahashi et al. 2018; Furukawa et al. 2020; Hellweg et al. 2023). Importantly, current research indicates that the biological effects of mixed radiation fields cannot simply be understood as a summation of the effects caused by individual radiation types (Staaf et al. 2012; Katugampola et al. 2024; Palmqvist et al. 2025). This emphasizes the critical relationship between the characteristic chromatin damage and the cellular radiation response (Falk and Hausmann 2020a, b; Hausmann et al. 2021a).

To add complexity, the biological effects of radiation are highly dependent on the nature of exposure. It is estimated that astronauts will accumulate a total dose of approximately 1 to 2 Gy during the round trip to Mars (Kiffer et al. 2019), a dose comparable to that experienced by first responders at Chernobyl (Shantyr et al. 1997). However, while astronauts will receive their protracted doses over a period of about 2 years, some of the Chernobyl liquidators were exposed to a similar dose within a short timeframe of several hours during a single acute exposure, leading to markedly different health consequences (Nelson 2016; Strigari et al. 2021).

**Fig. 3:**
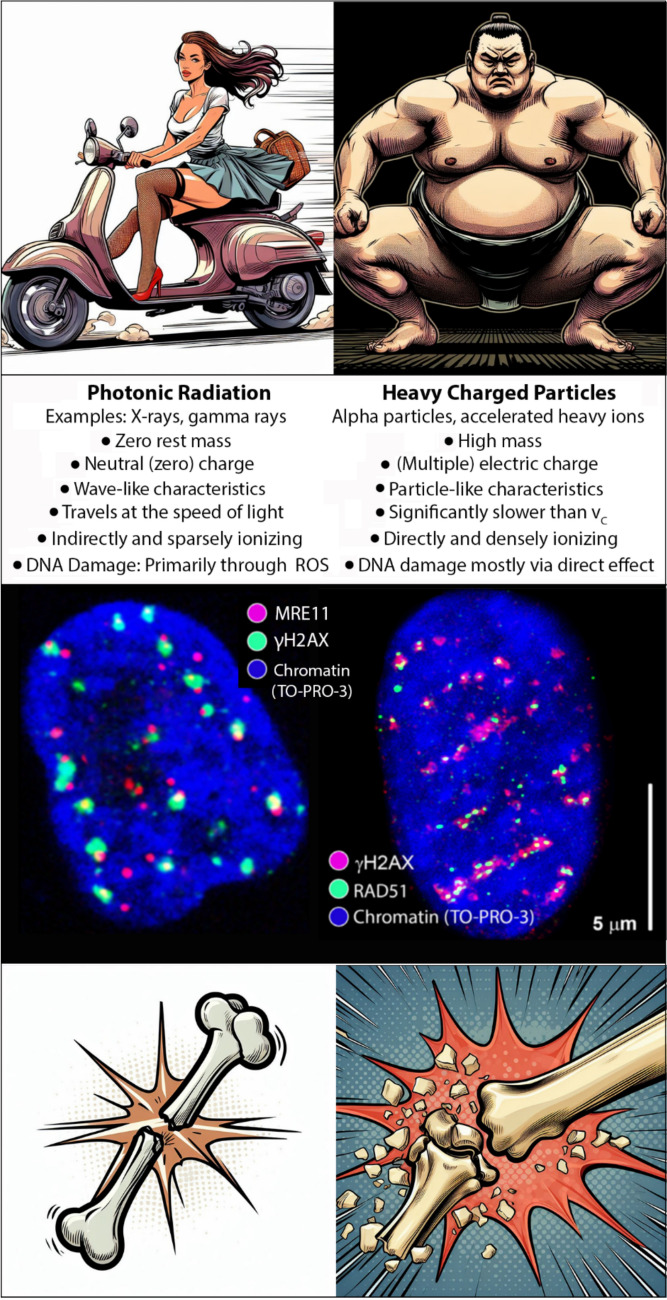
Basic classification of ionizing radiation into photon radiation (left) and heavy charged particles (right). The figure illustrates the fundamental characteristics of these types of ionizing radiation (IR) and their damaging effects on chromatin (lower panel). Proton radiation, despite its small mass, has physical characteristics similar to those of heavy charged particles; however, the type of damage to chromatin/DNA (spatially distributed simple double-strand breaks, DSBs) aligns more closely with that caused by photon radiation (as shown on the middle panel, left). Neutrons are slightly heavier than protons but carry no charge, similar to photons. Nonetheless, due to this characteristic, neutrons interact only with atomic nuclei, leading to the production of additional types of radiation; the resulting DNA damage corresponds to that caused by densely ionizing charged particles (the middle panel, right). The middle panel legend: the blue color in the middle panel represents chromatin (i.e., the cell nucleus), while red and green colors indicate two independent markers for double-strand breaks (DSBs) in DNA. DSBs are the most severe type of DNA damage. Green signals in the left nucleus and red signals in the right nucleus correspond to foci of γH2AX, indicating sites of phosphorylation of histone H2AX at serine 139 at DSB locations. The red signals in the left nucleus represent the MRE11 protein, which plays a crucial role at the initiation of DSB repair via both non-homologous end joining (NHEJ) and homologous recombination (HR) repair mechanisms. The green signal in the right nucleus visualizes foci of the repair protein RAD51, which acts later in DSB repair specifically at sites repaired via homologous recombination (HR) (and therefore occurs only in late S/G2 cells at DSB sites repaired via HR). Left nucleus: the maximum image created from the superposition of individual confocal slices of a human skin fibroblast nucleus exposed to 1 Gy of gamma radiation (^60^Co, 1 Gy/min) and visualized using immunofluorescence confocal microscopy 1 h post-irradiation. Approximately 21 γH2AX foci are visible, predominantly colocalizing with MRE11 protein foci and spatially separating from each other. Right nucleus: the same experiment as the left nucleus, but exposed to heavy charged ions (^15^N) with a high linear energy transfer (LET) of 180 keV/μm and an energy of 13.1 MeV/n (Jezkova et al. 2018; Falk and Hausmann 2020a) at the same dose. Most γH2AX and RAD51 foci accumulate along six linear trajectories corresponding to the paths of six ^15^N ions. The γH2AX and RAD51 foci here form complex clusters, indicating the creation of complex and multiple DSBs, which are lesions that cells struggle to repair; often, these can be lethal. Foci of γH2AX and RAD51 located outside the linear trajectories of the particle penetration channels denote DSB lesion sites created by secondary (delta) electrons ejected from surrounding atoms by the primary penetrating ion. The distribution of these foci (i.e., the trajectories and range of delta electrons) varies according to the physical characteristics of the specific ionizing radiation (Jezkova et al. 2018; Zadneprianetc et al. 2022). The bottom panels metaphorically compare double-strand breaks (DSBs) generated by low-LET (left panel) and high-LET (right panel) radiation to bone fractures. Low-LET radiation causes simple DSBs, akin to straightforward bone fractures that can be easily treated with a plaster cast. In contrast, high-LET radiation primarily results in multiple and complex DSBs, similar to splinter fractures with numerous small bone fragments that require more intricate surgical intervention for healing. Thus, the metaphorical illustrations highlight not only the different nature of DNA damage caused by low-LET and high-LET radiation but also the varying requirements for repairing these lesions, as well as the distinct risks of misrepair, such as the loss of one or more small bone fragments (deletion) or their incorrect reassembly (translocation, inversion).

Setting aside the almost philosophical debate about the dual wave-particle nature of microscopic objects, we can primarily categorize IR into two types based on their physical interactions and biological effects: photon radiation and particle radiation (Fig. 3) (Baatout 2023):

1. X-rays and gamma rays are both forms of photonic radiation. These types of radiation are typically classified based on their production mechanisms or energy (if the production mechanism is indeterminate, such as in astronomical contexts). X-rays are produced when accelerated electrons collide with the electron shells of target atoms. The passing electrons transfer their energy to the electrons in the target material, resulting in the excitation of these electrons to higher energy levels. Subsequently, these electrons return to lower energy levels (de-excitation), emitting energy in the form of photons, known as characteristic X-rays. Simultaneously, bremsstrahlung radiation is generated due to changes in the trajectory of the electrons as they pass through the electron shells of the target atoms (Kostin and Shipatov 1999; Baatout 2023). It is important to emphasize that bremsstrahlung radiation can be generated by all charged particles, not just electrons, though in much lower amounts due to their mass. Gamma radiation, in contrast, is emitted by the decay of unstable radioactive isotopes. Photons generally have zero rest mass, zero electric charge, and travel at the speed of light (Fig. 3). Due to these properties, they ionize their surroundings only indirectly (no electric charge) and “sparsely” (high speed of propagation), which is reflected in their high ability to penetrate matter (both air and biological tissues) while possessing a lower capacity to cause chromatin/cellular damage compared to accelerated particles (Talapko et al. 2024).
2. Energetic particles can be divided into two primary categories: charged and uncharged. Charged particles can further be categorized into light and heavy particles. Light charged particles typically include accelerated electrons and positrons. Heavy charged particles primarily consist of alpha particles and accelerated heavy ions (Schardt et al., 2010). However, while protons are classified within this category based on their physical properties (hadrons), they should be regarded as a distinct group in the context of this paper due to their unique effects on DNA and biological systems, which are significantly different from those of alpha particles and accelerated ions. Uncharged particles are represented solely by neutrons, excluding neutrinos, which have negligible mass and are not considered within the scope of this discussion due to their zero relevance to human health. Like photon radiation, neutrons are classified as indirectly ionizing radiation, as discussed further below.

### Neutron radiation: unique characteristics and biological implications

Neutron radiation represents a specific category of radiation due to its unique properties that, in some respects, resemble photon radiation while, in others, align more closely with beams of heavy charged particles. The high-energy neutrons constitute a significant component of the radiation produced by atomic weapons and nuclear reactors (Von Halban et al. 1939). Along with protons, alpha particles, accelerated ions, and gamma photons, neutrons are also part of primary cosmic radiation (Friedlander 2012).

From a physical perspective, neutrons can be classified as “particles” that, unlike accelerated charged particles, carry no electric charge. Consequently, the neutrons can interact only with the nuclei of surrounding atoms, leading to indirect ionization of the surrounding matter. Due to this mechanism of interaction, neutrons can penetrate materials as easily as photons despite having a non-zero mass, exhibiting similar energy deposition profiles (IARC Monogr Eval Carcinog Risks Hum. 2000). However, depending on their energy, neutrons can also exhibit exceptionally high relative biological effectiveness (RBE), reaching values comparable to those of alpha particles and heavy ions (Baatout 2023). The most hazardous neutrons are those with energies between 100 keV and 2 MeV, known as “fast neutrons,” which can be up to 20 times more dangerous (w_R_=20) than photon radiation (Stricklin et al. 2021).

This significant risk posed by neutrons in this energy range is attributed to a balance between sufficiently high energy for effective interactions with matter and excessively high energy which, accompanied by rapidly moving neutrons, drastically reduces the effectiveness of those interactions. Additionally, the ability of neutrons to interact directly with atomic nuclei is significantly associated with their capacity to activate surrounding materials, which then become sources of ionizing radiation themselves. This factor must be accounted for during shielding design and in the construction of nuclear reactors (Stricklin et al. 2021).

### Accelerated charged particles

Accelerated charged particles generally ionize their surrounding environment directly due to their charge and, relative to their mass, move more slowly than energetic photons (Fig. 3). The density of ionization, defined as linear energy transfer (LET; keV/μm), strongly depends on the charge and velocity of the particle (Baatout 2023), where the velocity is determined by the particle’s kinetic energy.

The charged particles, thus, transfer their energy to the surrounding matter (such as tissue) continuously as they decelerate, resulting in a fundamentally different dose depth profile compared to photon and neutron radiation (Durante 2019; Durante et al. 2021). This energy deposition as a function of material (tissue) depth is described by depth-dose curves (Durante 2019; Baatout 2023).

A comparison of these curves for different types of radiation reveals that energetic photons deposit most of their energy at the surface of the tissue, whereas charged particles release their energy mostly at a specific depth, which is determined by the particle’s kinetic energy and other parameters. While photons can lose their energy completely during discrete stochastic events, such as Compton scattering—which may be accompanied by the emission of lower-energy photons—charged particles are continuously slowed down as they traverse tissue, eventually coming to a stop. As penetration depth increases, LET of the particle continues to grow until the particle releases the majority of its energy just before the end of its trajectory, at the so-called Bragg Peak (Surdutovich and Solov’yov 2017; Solov’yov et al. 2024; Guan et al. 2024). Hence, in contrast to photon radiation, with appropriate particle energy selection, it is possible to target energy deposition more precisely within the tumor volume. The tissue before and especially beyond the Bragg Peak is irradiated significantly less compared to the Bragg Peak region, which can be advantageously utilized for certain tumor types, especially those localized near critically important organs and structures (Pennock et al. 2023). In clinical practice, the energy spectrum of a polyenergetic particle beam can be modulated such that different particles exhibit their Bragg peaks at varying depths within the tissue. This results in a spread-out Bragg peak (SOBP), which accurately covers the tumor volume (Darafsheh et al. 2021).

The electrons and protons carry an electric charge. Although electrons are approximately 7280 times lighter than alpha particles and protons are about four times lighter, the energy deposition characteristics of these ‘lighter’ charged particles resemble those of alpha particles and heavy charged ions (Mott and West 2021; Pennock et al. 2023). However, the ionization density of electrons and protons is relatively low, resulting in radiation-weighting factors (w_R_) and relative biological effectiveness (RBE) values that are similar to those of photon radiation (Morgan and Vajuhudeen 2020; Baatout 2023). Consequently, rapidly advancing proton therapy offers more precise targeting of radiation energy to tumor volumes compared to standard photon radiation therapy, although the overall efficacy in killing tumor cells is not markedly greater.

Heavy charged particles, due to their mass and charge, experience significant slowing as they traverse matter. This deceleration is reflected in both the previously discussed dose depth profiles and the high, progressively increasing ionization density along their path (linear energy transfer, LET) (Schaub et al. 2020; Durante et al. 2021; Baatout 2023). Similar to light charged particles, heavy charged particles also provide more precise targeting of radiation dose to the tumor. However, compared to light particles and photons, they cause much more severe damage to chromatin (Fig. 3) and cells (Lopez Perez et al. 2016; Jezkova et al. 2018; Zadneprianetc et al. 2022). Both of these properties are strategically utilized by the developing field of hadron therapy using carbon ions and potentially other ions as well (Saunders et al. 1985; Ebner and Kamada 2016; Dokic et al. 2016; Ebner et al. 2021).

The differences in the physical characteristics of the aforementioned types of radiation and their specific mechanisms of interaction with matter are reflected in the nature of the chromatin damage they induce (Fig. 3). This relationship will be discussed in the next chapter.

## A general mechanism of radiation-induced biological effects

The ability of ionizing radiation (IR) to kill cells and induce various acute and late syndromes, including tumorigenesis, was recognized shortly after Wilhelm Conrad Röntgen discovered X-rays in 1895. Investigating why irradiated cells die, how radiation damage leads to the development of tumors, and how to effectively harness it for tumor elimination has since become a central focus of radiobiological research (Baatout 2023).

When considering the biological impacts of irradiation from an energy perspective, it is surprising to find that the energy transferred to tissue from 1 Gy of radiation is approximately equal to the energy obtained from drinking a cup of espresso (Falk and Falkova 2020). However, even such a small deposited energy exceeds the threshold dose (approximately 0.6 Gy) for inducing the hematopoietic form of acute radiation syndrome (ARS) (Baatout 2023). At ten times this dose, or 10 Gy, we enter a range where exposure levels are so high that no individuals are likely to survive a single whole-body exposure (Talapko et al. 2024). A dose of 10 Gy corresponds to 10 J/kg, meaning that an average adult (approximately 80 kg) would absorb 800 J. For comparison, warming 1 liter of water by 1 °C requires 4180 J, which is roughly four times greater. Therefore, while a dose of 10 Gy would reliably prove fatal, it would only increase the body’s temperature by a mere 0.002 °C. Hence, the actual energy magnitude transferred by radiation to tissue does not explain the ability of IR to effectively kill cells.

It is evident that the mechanism of energy transfer plays a crucial role, and it is clear that there must be a critical target within the cell that is particularly sensitive to the effects of IR. It is now widely accepted that this target is the DNA within the cell nucleus, although studies have also indicated a potentially dominant role of protein dysfunction that facilitates the elimination of reactive oxygen species (ROS) in various bacterial strains (Daly et al. 2007; Krisko and Radman 2013; Falk and Falkova 2020). IR induces a variety of different DNA lesions, among which the most dangerous are double-strand breaks (DSBs) (Tchurikov et al. 2022). DSBs can be generated also by some (therapeutic or warfare) chemicals (Rittich et al. 2004; Inturi et al. 2014; Amarh and Arthur 2019; Jan et al. 2020) but IR is their most efficient inducer. Theoretically, even a single DSB can lead to cell death if left unrepaired or can cause tumorigenic transformation if repaired incorrectly. In summary, it is the transfer of radiation energy in the form of large quanta (corresponding to a single photon or particle) that impacts sensitive targets (DNA) which accounts for the high capacity of ionizing radiation to damage and kill cells.

## DNA double strand break (DSB) induction by sparsely and densely ionizing radiation

The danger of double-strand breaks (DSBs) lies in their ability to simultaneously sever both strands of the DNA molecule at the same location, leading to complete disintegration. A DSB can also occur as a result of two single-strand breaks (SSBs) affecting opposing DNA strands in close proximity, meaning they do not necessarily have to be directly opposite each other (Pawelczak et al. 2011). This can lead to the generation of DNA ends with different characteristics. If the resulting free DNA ends are not promptly ligated through repair mechanisms, they can become separated and misrejoined, either with ends from other chromosomes or incorrect regions of the same chromosome. This can lead to chromosomal translocations and other chromosomal aberrations (Falk et al. 2010). The frequency of translocations, dicentric chromosomes, and ring chromosomes correlates with the absorbed dose of IR, positioning these aberrations as important biomarkers for estimating absorbed doses, particularly useful in large-scale radiation accident situations where precise dosimetric data are usually not available (Herate and Sabatier 2019).

The hazards associated with DSBs induced by IR can also be attributed to additional factors: IR creates complex and multiple breaks, often in combinations (Davis and Chen 2014; Jezkova et al. 2018; Mavragani et al. 2019; Zadneprianetc et al. 2022). The complex DSBs are defined as lesions where DSBs spatially colocalize with other types of DNA damage, such as oxidative base damage (Kumar et al. 2023). Multiple DSBs occur when several DSBs and/or SSBs cluster within close proximity in the DNA molecule, potentially leading to local fragmentation of chromatin. This is broadly comparable to chromothripsis, as seen in conditions such as myelodysplastic syndromes, although the mechanisms underlying chromatin fragmentation differ (Pagáčová et al. 2014; Abáigar et al. 2016; de Groot et al. 2023; Erenpreisa et al. 2023). The complex and multiple DSBs can be challenging to repair or may not be repairable at all (Asaithamby et al. 2011; Lorat et al. 2016; Jezkova et al. 2018; Roobol et al. 2020). As such, these lesions are considered critical in terms of the biological effects of IR and play a decisive role in determining the fate of the irradiated cell (Falk and Hausmann 2020b; Solov’yov et al. 2024). Moreover, the DSBs caused by IR generally cannot be ligated without prior enzymatic “cleaning” of DNA ends, which, depending on the repair mechanism, can result in the creation of small or extensive deletions/insertions at the DSB sites (Shibata and Jeggo 2020).

IR induces DSBs through two mechanisms: direct and indirect. The early observations following the discovery of IR showed that cells in a hypoxic environment survive significantly better at a given dose of IR than cells with normal oxygen saturation (Bristow and Hill 2008; Bouleftour et al. 2021). Hypoxia continues to complicate radiotherapy today, often rendering it infeasible, as it makes tumor cells much more resistant to IR compared to normal cells, even considering the higher proliferation rate of tumor cells (Bristow and Hill 2008; Bouleftour et al. 2021). These findings directed scientific attention to the role of reactive oxygen species (ROS) (Halliwell et al. 2021) in the mechanism of radiation-induced DNA damage. ROS are produced via the radiolysis of water within cells and, upon contact with the DNA molecule, generate single-strand breaks (SSBs) (Srinivas et al. 2019; Solov’yov et al. 2024). Consequently, the formation of DSBs requires the simultaneous attack of two ROS in close proximity, which, given the amount of ROS produced, is not uncommon. This mechanism primarily causes DNA damage from photon radiation, where the indirect effect contributes to overall DNA damage by approximately 70–80 % (Falk and Falkova 2020).

In the case of heavy charged particles, on the other hand, DNA is damaged primarily by the traversing particles themselves (Falk and Falkova 2020). This mechanism is referred to as the direct effect of IR. Therefore, the lethal effect of this type of irradiation is much less dependent on ROS compared to photon radiation, and consequently, on tissue oxygen saturation (Hill et al. 2022). DSBs can be generated from the passage of a single particle, unlike the indirect effect which requires concurrent attacks from two ROS at a specific location in the DNA. Thus, radiotherapy using accelerated heavy ions can be effective even in the case of hypoxic tumors (Hill et al. 2022), of which there are many. The killing capacity of densely ionizing particles is also less influenced by the genetic background of tumor cells, such as the functionality of the p53 protein (Yamakawa et al. 2008; Mori et al. 2009).

## LET and the mechanisms of radiation-induced DNA/chromatin damage

Ionizing radiation (IR) poses a specific risk due to its ability to induce DSBs, which can severely compromise cellular integrity, as already mentioned. The mechanisms through which IR affects biological systems primarily involve the deposition of energy and the resulting distribution of ionization events at the microscale (Fig. 3). Exposure to different radiation types results in a distinct distribution of DSBs within chromatin, different character (multiplicity, complexity) of DSBs, and consequently ability of cells to repair these lesions (Fig. 3) (Jezkova et al. 2018; Zadneprianetc et al. 2022). As photon radiation primarily damages DNA only indirectly through the generation of reactive oxygen species (ROS) in its proximity, many photons are required to generate high enough numbers of DSBs or even complex or multiple DSBs (SSBs in close proximity) to kill the cell. Moreover, the photons interact with matter infrequently, and the nature of these interactions is stochastic, allowing a photon to lose all of its energy in a single interaction. Consequently, ROS, secondary emitted electrons, and DSBs are randomly distributed throughout the entire volume of the nucleus following exposure to photon radiation (Fig. 3). Thus, DSBs tend to manifest as spatially dispersed, simple lesions that are relatively easy to repair. When absorbing a dose of 1 Gy, an average human cell nucleus can exhibit approximately 25 to 35 repair foci marking individual DSB sites (Vicar et al. 2021).

Heavy ion radiation, on the other hand, induces a completely different type of chromatin damage (Fig. 3). To deliver the same given dose as in the case of photon radiation (e.g., 1 Gy in Fig. 3), only one or several particles are required to cross the nucleus (as depicted in Fig. 3) (Jezkova et al. 2018; Zadneprianetc et al. 2022). The particles traversing the cell nucleus create DSBs within the DNA molecule mostly by themselves and secondary electrons, meaning that a single interaction with the DNA is sufficient to induce a DSB. The charged particles transfer their energy gradually, which generates multiple DSBs and additional types of DNA damage along their trajectory (Fig. 3) (Jezkova et al. 2018; Hausmann et al. 2021a; Danforth et al. 2022; Zadneprianetc et al. 2022). Moreover, the high density of ionization caused by particles results in significant ROS production and accumulation in a narrow ionization channel along the particle’s path (Solov’yov et al. 2024). This high concentration of radicals in a limited volume causes some to recombine into benign molecules; nevertheless, a substantial number of highly reactive molecules remain available to create a considerable number of DSBs (Le Caër 2011). Additional ROS are generated by secondary (delta) electrons that escape beyond the core of the ionization channel and contribute to both clustered and isolated DSB formations (Datta et al. 2012). Due to the close proximity of these DSBs and their coexistence with other types of DNA damage, many lesions caused by exposure to densely ionizing charged particles exhibit characteristics of multiple and/or complex DSBs, which are by principle particularly difficult or impossible for the cell to repair (Jezkova et al. 2018; Mladenova et al. 2022b, a). The passage of a particle through chromatin generates a shock wave akin to the pressure wave produced by an explosion (Solov’yov et al. 2024), which further damages the chromatin and disperses reactive oxygen species (ROS) over extended distances from the particle’s trajectory. As a consequence of all processes mentioned above, significant fragmentation of the chromatin occurs locally. This fragmentation not only poses challenges for repair but also has been shown to ‘actively’ inhibit certain DNA repair mechanisms, however, with contradictory results on what mechanism is inhibited or preferred (Schipler and Iliakis 2013; Koltsova et al. 2019; Nickoloff et al. 2020; Li and Wang 2023; Hu et al. 2024). Consequently, the cell’s ability to address chromatin damage inflicted by densely ionizing particles is further compromised.

The complex and multiple DSB lesions carry a high risk of genomic damage; however, due to their generally lethal nature, the likelihood of initiating secondary malignancies after hadron therapy is paradoxically lower than that following photon radiation therapy, at least according to some studies (Newhauser and Durante 2011; König et al. 2020). Utilizing accelerated heavy ions for therapy thus offers a significantly more effective means of eradicating even hypoxic or otherwise radioresistant tumors while providing better protection to surrounding tissues, due to both more precise targeting of radiation energy deposition within the tumor volume and lower risks of inducing secondary malignancies.

**Fig. 4:**
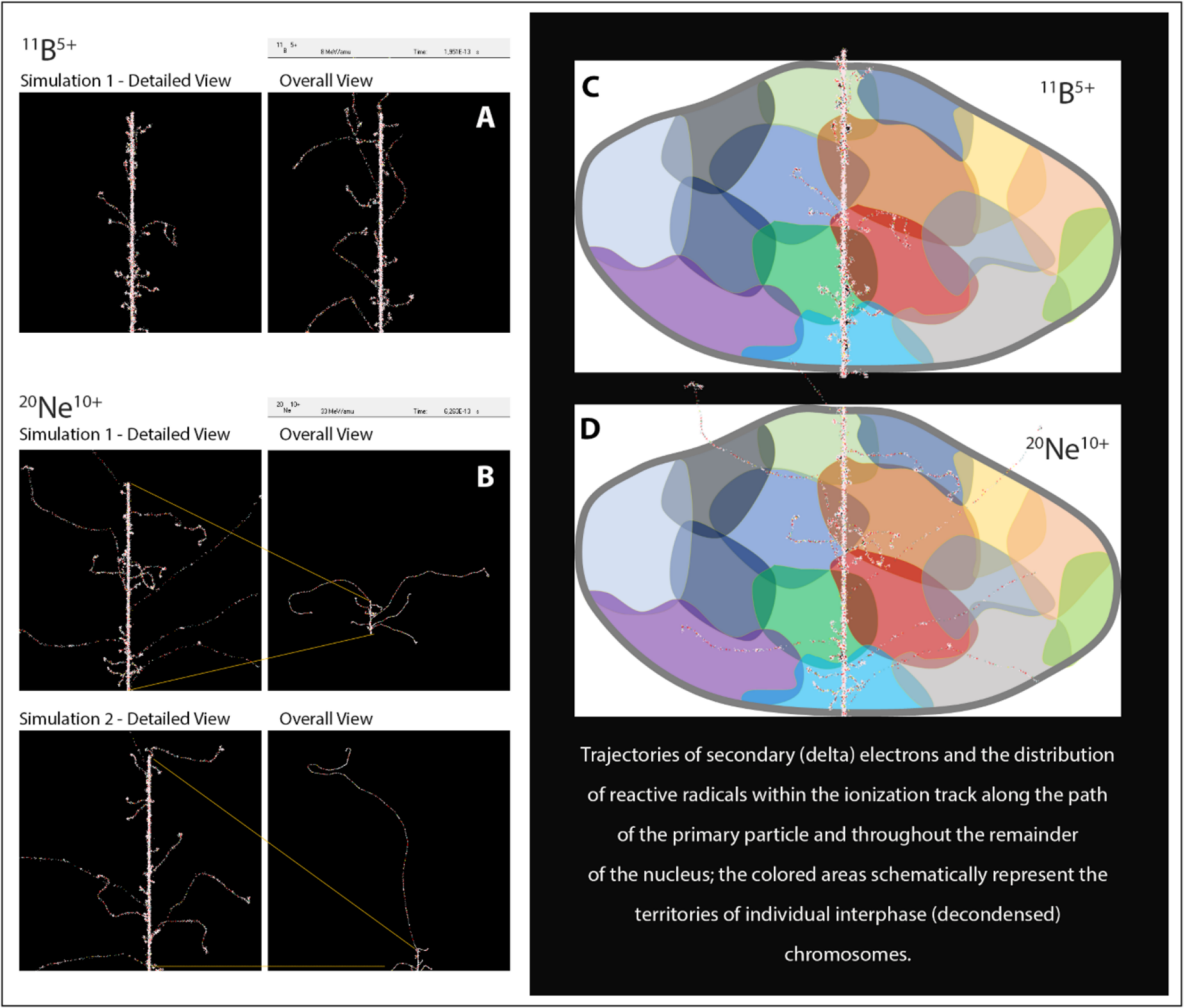
Simulation of trajectories and distribution of secondary (delta) electrons and reactive radicals generated by ^11^B^5+^ and ^20^Ne^10+^ ions with similar LET values and approximately low energy (^11^B^5+^: LET 148.3 keV/μm, 7.5 MeV/nucleon, and ^20^Ne^10+^: LET 170.9 keV/μm, 33.9 MeV/nucleon). The left images in the left panels (**A**, **B**) display enlarged sections (marked by yellow dashed lines) of the right images, where the scale is chosen to show the entire paths of the most distant secondary electrons. The magnification of the images for the ^11^B^5+^ and ^20^Ne^10+^ ions is the same. Simulations were performed using the RITRACK software provided by NASA (Jezkova et al. 2018) (https://software.nasa.gov/software/MSC-25937-1). The right panels (**C**, **D**) illustrate how differences in the distribution and range of delta electrons affect DNA damage within the cell nucleus. For the ^11^B^5+^ ions, the main ionization channel is somewhat narrower than that of the ^20^Ne^10+^ ions, which also have a shorter range of delta electrons. This suggests that these two different ions induce DNA damage in slightly different ways, despite being accelerated to similar LET and roughly similar energy levels. Specifically, the ^11^B^5+^ ions concentrate similar energy in a narrower channel core than that generated by the ^20^Ne^10+^ ions, which reduces their ability to induce complex double-strand breaks (DSBs). Furthermore, the ^20^Ne^10+^ ions, due to their greater range of delta electrons, can damage a wider variety of chromosomes, potentially leading to more complex genomic rearrangements (panels **C**, **D**) (Jezkova et al. 2018)

It is important to note that the multiplicity, complexity, and reparability of DSB lesions are significantly influenced by both the energy and linear energy transfer (LET) of the particle. However, our recent findings indicate that the situation is more intricate, as the charge and other parameters of the particle also play a substantial role in determining the characteristics of the traversal channel and the microdosimetric distribution of secondary (delta) electrons emitted from the surrounding medium (Santa Cruz 2016; Jezkova et al. 2018; Missiaggia 2024). Consequently, the distribution of DNA damage within the cell nucleus and its reparability can vary among different ions, even when they are accelerated to exhibit similar energy and LET (Fig. 4) (Jezkova et al. 2018). This finding may be important in the search for potentially beneficial ions for radiotherapy, as well as for more accurately assessing the health risks posed to astronauts from exposure to cosmic radiation.

However, higher LET does not always equate to more effective tumor elimination, even though chromatin damage in irradiated cells increases. The relative biological effectiveness (RBE) of radiation increases with LET up to approximately 100 keV/μm, beyond which the phenomenon known as the overkill effect occurs (Sørensen et al. 2011). The essence of the overkill effect is that even a single particle with an LET of about 100 keV/μm can induce DNA damage that ultimately results in cell death. Consequently, any further increase in LET does not enhance tumoricidal efficacy but instead reduces the number of irradiated cells, as a given dose is imparted by a lower number of particles. In other words, excessive energy is utilized to kill individual cells, thereby diminishing the energy available for the destruction of additional cells (Mehnati et al. 2005). As a result, in the LET range above 100 keV/μm, the RBE begins to decrease with increasing LET.

## Radiation damage in the context of structurally and functionally distinct chromatin domains

Coding and transcriptionally active regions of DNA are not randomly distributed throughout the genome; instead, they are organized into functional clusters (Caron et al. 2001). Consequently, chromatin exhibits a non-random three-dimensional (3D) organization within the cell nucleus, forming domains with specific structural features and functions (Cremer et al. 1982, 2015; Bártová et al. 2001; Lukásová et al. 2002; Falk et al. 2002; Kozubek et al. 2002; Amrichová et al. 2003; Misteli 2020). One prominent example includes euchromatin and heterochromatin, where, in simplified terms, euchromatin is considered genetically active, while heterochromatin is deemed inactive (Allshire and Madhani 2018; Erenpreisa et al. 2021). These functional distinctions align with structural differences—euchromatin is significantly less condensed compared to heterochromatin, which is tightly packed and associated with various proteins that mediate this condensation (Bannister et al. 2001; Cheutin et al. 2003; Fischer et al. 2023).

**Fig. 5:**
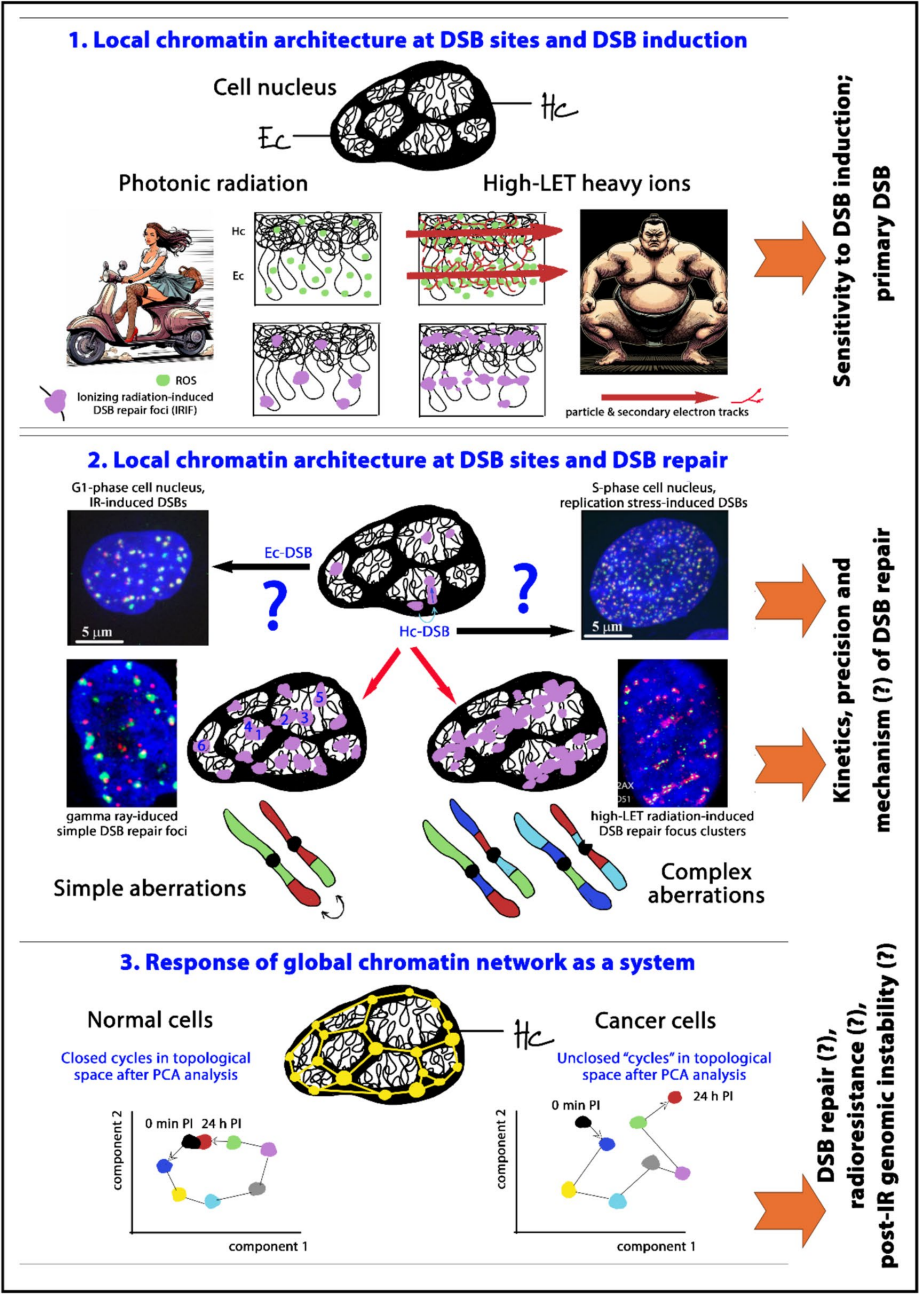
Fundamental aspects of radiation-induced DNA damage and cellular DNA damage response influenced by interaction between incident ionizing radiation (IR) and chromatin architecture. Top Panel: Two primary types of chromatin—euchromatin (Ec) and heterochromatin (Hc)—exhibit distinct structural characteristics; Ec is less condensed and more hydrated than Hc, which influences their sensitivity to DNA double-strand break (DSB) induction by ionizing radiation (IR). The high abundance of heterochromatin binding proteins in Hc (e.g., HP1) provides some protection against reactive oxygen species (ROS) generated by radiation, resulting in fewer DSBs in Hc compared to Ec. This difference is particularly pronounced with low linear energy transfer (LET) radiation, such as gamma rays, which primarily inflict damage through ROS. In contrast, high-LET radiation particles directly induce DNA damage, largely independent of chromatin structure, leading to a greater number of DSBs in Hc due to the higher density of DNA targets per unit volume within Hc. Additionally, the more hydrated nature of Ec contributes to increased ROS production in this chromatin type. Since most ROS have extremely short lifespans, this results in more DSBs being generated in Ec. Middle panel illustrates how chromatin architecture at the sites of DNA DSBs and the type of incident radiation influence DSB repair processes**:** DSBs are typically repaired at their sites of formation. However, for DSBs localized in heterochromatin (Hc), mobility increases due to the decondensation of the damaged Hc domain, which is essential for the formation of repair complexes (ionizing radiation-induced foci, IRIF). Specifically, some repair proteins can penetrate heterochromatin before decondensation, while others do so only afterward. This differential access regulates the spatiotemporal involvement of repair proteins at DSBs, thereby guiding the formation of repair complexes influenced by the local chromatin architecture surrounding individual DSBs. Hence, it appears that the pre-irradiation local chromatin architecture at DSB sites, as well as its modifications during DSB repair processes, significantly contribute to the selection of specific repair mechanisms (Baldock et al. 2015; Falk and Hausmann 2020b)., thus determining the kinetics and accuracy of repair. This claim is supported by our preliminary results indicating distinct morphology of repair foci γH2AX and 53BP1, depending on their location in Ec or Hc, while it is known that repair mechanisms in Hc differ from those in Ec (Hc tends to favor homologous recombination or ATM-dependent non-homologous end joining (NHEJ), although the situation is considerably more complicated and unclear). Differences in the morphology of DSB repair foci are especially evident for DSBs generated in G1-phase nuclei by gamma radiation (predominantly in Ec) and primarily repaired via NHEJ when compared with DSBs arising from replication stress that are repaired through homologous recombination (Falk et al. 2018). Repair of DSBs in Hc is inherently linked to decondensation of the damaged Hc domain, followed by reconstruction of chromatin architecture to its original pre-irradiation form (Falk et al. 2007). This has important consequences: (1) repair in heterochromatin is significantly slower than in Ec and carries a risk of epimutations (i.e., aberrations in chromatin architecture that persist even after successful rejoining of DSBs); (2) DSBs protrude from Hc into surrounding nuclear subspaces with lower chromatin density. Protruding Hc DSBs and Ec DSBs already present in these restricted spaces can later interact, leading to the formation of secondary (repair-induced) clusters of DSBs (Falk et al. 2014b, which can ultimately result in chromosomal translocations. Importantly, the specific location where a given Hc DSB protrudes is determined by the local chromatin architecture within its vicinity. Therefore, the probability of translocation between two specific genetic loci is not solely defined by their (non-random) spatial (3D) distance within the cell nucleus, as was previously believed, but also by the architecture (texture) of the chromatin between the respective DSBs (Falk et al. 2010; Falk and Hausmann 2020b). In illustration figure (the middle panel), there are six repair foci labeled as 1 through 6. The spatially closest repair foci are 1 and 2; however, they are separated by a heterochromatic barrier due to the local chromatin architecture and positioned such that, during the repair-associated decondensation, they protrude into different nuclear subcompartments. Thus, the probability of chromosomal translocation is significantly higher between foci 1 and 4 or 2 and 3, even though these DSBs are originally more distant from each other than DSBs 1 and 2. For completeness, the chromosomal translocations can occasionally occur even between very distant DSBs, such as between 2 and 5 or 1 and 6 (Finn et al. 2017). In these cases, there may be a higher, albeit very slight, probability of interaction between DSBs 2 and 5, since DSBs 1 and 6 are again separated by a heterochromatic barrier. The secondary clusters of DSBs, formed through repair processes, explain how chromosomal translocations can arise, occasionally involving more than two chromosomes, in cells exposed to sparsely ionizing radiation. In the case of densely ionizing radiation, primary multiple clusters of DSBs, resulting from chromatin fragmentation due to locally significant energy deposition in a very confined space along the particle’s trajectory, predominantly contribute to the formation of translocations. In contrast to gamma radiation exposure, irradiation with densely ionizing particles thus leads to the creation of multiple, highly complex DSB clusters even in the absence of repair processes. Consequently, chromatin fragmentation results in complex chromosomal aberrations. Importantly it also likely inhibits some repair mechanisms, possibly NHEJ due to the inability of Ku proteins to bind to short DNA fragments (although the situation remains unclear). (possibly NHEJ; see the main text). Together, our results support the scenario where the local architecture of chromatin at DSB sites, in conjunction with the physical characteristics of incident radiation, significantly influences various aspects of DSB repair through multiple mechanisms. Bottom panel highlights the role of the entire chromatin network in responding to radiation-induced damage**:** the entire chromatin network also responds to cell irradiation as a functional system (Erenpreisa et al. 2023; Weidner et al. 2023; Falk et al. 2025a, b)﻿. The nano-level experiments using single-molecule localization microscopy (SMLM) are beginning to reveal that irradiation alters the nanotopology of both repair foci and heterochromatin network marked by the epigenetic modification H3K9me3 (Erenpreisa et al. 2023; Schäfer et al. 2024; Falk et al. 2025a, b). Notably, there is a fundamental difference in the responses of normal and various tumor cells. When examining changes in heterochromatin architecture as movements in topological space following PCA analysis over time after irradiation (illustrative only plots; 0 min PI “control” to 24 hours post-irradiation, when most DSBs are repaired), it can be seen that normal cells exhibit closed cycles, with heterochromatin architecture returning to its original state after DSB repair is completed. In contrast, tumor cells do not show this reversion, and their cycle remains open (Weidner et al. 2023; Falk et al. 2025a, b). Persistent disrupted chromatin architecture may contribute to or promote genetic instability in tumor cells, which is observed to be heightened after irradiation (Wang et al. 2018). For example, this may lead to the activation of transposons (Sun et al. 2020; De Oliveira et al. 2021; Du et al. 2023). Additionally, radioresistant cells appear to exhibit less pronounced changes in chromatin network architecture after irradiation compared to radiosensitive cells.

Thus, it is critical to explore how the mechanisms of interaction with matter, known for sparsely and densely ionizing types of radiation, influence radiation-induced damage within these structurally and functionally distinct chromatin domains. Our previous studies (Falk et al. 2008a, b) have shown that heterochromatin-binding proteins can sequester reactive oxygen species (ROS), providing some degree of protection to DNA from the indirect damage caused by photon radiation. Conversely, euchromatin tends to be more hydrated than its condensed counterpart, leading to increased ROS production following irradiation. As most ROS are short-lived, they mainly damage DNA in close proximity to their site of formation, i.e., mostly in euchromatin (Falk et al. 2008b). Therefore, the transcriptionally active euchromatin, which houses crucial proto-oncogenes and tumor suppressor genes, is more vulnerable to damage from photon radiation compared to heterochromatin (Falk et al. 2008b; Takata et al. 2013). On the other hand, the condensed architecture of heterochromatin complicates the repair of double-strand breaks (DSBs), as discussed later and summarized in Fig. 5 (Falk et al. 2007, 2010, 2014c; Ayoub et al. 2008; Noon et al. 2010; Goodarzi et al. 2010; Goodarzi and Jeggo 2012; Baldock et al. 2015).

In contrast, when it comes to densely ionizing heavy particles, neither the proteins bound to chromatin nor other structural features provide any protection against the direct action of a passing particle (Falk et al. 2014b; Falk and Hausmann 2020b). Unlike photon-irradiated cells, heterochromatin tends to sustain more damage than euchromatin because of its density, which offers more DNA targets per unit volume (Dos Santos et al. 2014; Lorat et al. 2016; Falk and Hausmann 2020b). Consequently, densely ionizing charged particles induce more complex DNA lesions in heterochromatin than in euchromatin, with the repair of these already challenging lesions being further complicated by the heterochromatin structure (Dos Santos et al. 2014; Lorat et al. 2016; Falk and Hausmann 2020b). It should also be noted that euchromatin suffers substantial damage when traversed by heavy charged particles (Figs. 3 and 5). Moreover, damaging heterochromatin may lead to serious but not fully understood consequences for the cell. Despite being inactive from a classical genetics perspective, heterochromatin likely plays a crucial role in the spatial organization of the cell nucleus and forms regulatory networks, enabling the nucleus to respond to various stressors as a coordinated functional system (Erenpreisa et al. 2021, 2023). As detailed later, our recent studies showed that heterochromatin network responses differently to irradiation in normal and various tumor cells as discussed later (Fig. 5, bottom panel) (Falk et al. 2025a).

IR can also induce not only changes in DNA sequence, i.e., mutations, but also epimutations (Falk et al. 2014b; Ježková et al. 2014; Belli and Indovina 2020). As previously mentioned, chromatin must adopt a specific structure to function correctly. However, this structure changes both as a result of radiation damage and subsequently during repair processes. It is possible that while a cell may successfully rejoin free DNA ends, it may not restore the original structure of the chromatin at the site of the DSB. This may lead to altered expression of the affected chromosomal locus, disruption of the whole heterochromatin network, and potentially pathological manifestations, such as transposon activation (Sun et al. 2020; De Oliveira et al. 2021) and tumorigenesis (Kamstra et al. 2018; Siblini et al. 2021). If gametic cells are affected, these epigenetic changes may even be inherited (Amrenova et al. 2024), potentially occuring in several generations of unirradiated offspring of exposed parents (Amrenova et al. 2024). Given that densely ionizing heavy charged particles have a significantly more devastating impact on chromatin architecture than photon radiation, it can be reasonably expected that the risk of epimutations (in a broader sense) will be more pronounced following exposure to this type of ionizing radiation compared to photons, assuming the cells survive irradiation.

## Repair of DSBs within chromatin environment

Given the omnipresence of ionizing radiation and its significantly higher intensity on prehistoric Earth, the cells have evolved efficient mechanisms for repairing radiation-induced DNA damage over the course of evolution (Prorok et al. 2021). The homologous recombination (HR) (Li and Heyer 2008) and non-homologous end joining (NHEJ) (Chang et al. 2017) are two fundamental mechanisms for repairing DSBs in eukaryotic cells (Scully et al. 2019; Baatout 2023). NHEJ is a rapid repair mechanism in which free DNA ends, after enzymatic processing, are simply joined together. This makes NHEJ an essential and efficient repair pathway, especially in cells with large genomes, where the prompt reconnection of potentially extensive DSBs is critical to preventing dangerous chromosomal aberrations and cell death during mitosis. In human cells, the coding sequences of DNA account for approximately 2 % of the genome, and only a portion of these sequences directly or indirectly influences the balance of cell proliferation (Comfort 2015). The unrejoined DSBs or chromosomal aberrations can prevent the cell from entering mitosis or lead to improper segregation of genetic material (chromosomes) during cell division. The latter scenario is typically responsible for what is known as mitotic death, the most common type of death observed in cells exposed to IR (Firat et al. 2011). Alternatively, irradiated cells with unrepairable DSBs may enter senescence or survive in a cycling state with an unstable genome due to polyploidization (Bretscher and Fox 2016; Noda 2018). The polyploid cells are genetically unstable and pose a considerable risk for the initiation of new (radioresistant) tumor cell clones (Noda 2018). Until recently, senescent cells were thought to be safely inactivated. However, it has been shown that under certain conditions, even senescent cells can begin proliferating again (Afifi et al. 2023), thus representing a sort of genetic time bomb within the organism. Therefore, NHEJ can protect a cell from death while presenting a relatively low risk of mutating critical genomic sequences and transforming the cell.

HR, on the other hand, operates as a complex and slower process that, in most situations, can restore DNA integrity without altering genetic information (Li and Heyer 2008; Baatout 2023). The specific enzymes first degrade one strand of the double-stranded DNA molecule over long sequences on both sides of the DSB site. This process creates single-stranded DNA ends that subsequently seek homologous sequences on the sister chromatid of the chromosome, forming a triplex structure. Based on the genetic information recorded in the nucleotide sequence of the intact chromatid, the missing segments of the DNA strands are resynthesized and subsequently ligated (Li and Heyer 2008; Baatout 2023). HR allows for the repair of even very complex DSB lesions without creating mutations, but it is primarily limited to the late S and G2 phases of the cell cycle (Baatout 2023). However, homologous recombination (HR) can also become mutagenic and hazardous under certain conditions (Wells and Feschotte 2020), for instance when it functions improperly within heterochromatin, which is rich in repetitive sequences (Falk and Hausmann 2020b).

The repair of DSBs involves the accumulation of repair proteins at the sites of DSBs, leading to the formation of repair complexes, which can be visualized using fluorescently labeled antibodies and immunofluorescence confocal microscopy (Falk et al. 2007, 2008a; Sevcik et al. 2012, 2013; Jezkova et al. 2018; Pagáčová et al. 2019; Vicar et al. 2021) or super-resolution microscopy, such as single-molecule localization microscopy (SMLM) (Bach et al. 2017; Depes et al. 2018; Bobkova et al. 2018; Hausmann et al. 2021a; Weidner et al. 2023; Solov’yov et al. 2024). These localizations are referred to as ionizing radiation-induced foci (IRIF) (Fig. 3), which evolve in their composition and characteristics over time following irradiation. The techniques like immunofluorescence confocal microscopy have thus become crucial in radiation damage and DNA repair research because they allow for the study of the formation, “dissolution”, and nuclear distribution of IRIFs during repair processes in intact or even live cells. Furthermore, microscopy has provided valuable insights into the spatial and temporal organization of repair processes, which is essential for understanding the mechanisms of DSB repair across different chromatin domains and in response to various types of ionizing radiation. For more detailed information on the applications of microscopy and nanoscale imaging in radiobiological research and biodosimetry, we recommend referring to our previously published papers (Falk et al. 2007, 2008b, 2010; Sevcik et al. 2012, 2013; Bach et al. 2017; Jezkova et al. 2018; Bobkova et al. 2018; Vicar et al. 2021; Hausmann et al. 2021a; Weidner et al. 2023). It is important to note that advancements in optical super-resolution microscopy now allow us to achieve resolution at the level of individual molecules, for instance, through techniques such as SMLM (Hausmann et al. 2017, 2018, 2021b; Falk and Hausmann 2020a; Weidner et al. 2023). This approach enables researchers to study the organization of IRIFs in terms of their geometry and topology, as well as the repair processes of DSBs at the nanoscale (Bach et al. 2017; Bobkova et al. 2018; Dobešová et al. 2022; Weidner et al. 2023; Solov’yov et al. 2024; Schäfer et al. 2024; Falk et al. 2025a).

One of the first steps in the repair of DSBs is the phosphorylation of histone H2AX at serine 139 across extensive regions of chromatin (up to 2 Mb in humans) surrounding the DNA break. This phosphorylated histone, denoted as γH2AX, serves as both a signal for the presence of DSBs and facilitates the reconfiguration of chromatin structure into a conformation necessary for repair, allowing for the coordinated recruitment of a range of proteins to the damage site (Rogakou et al. 1998, 1999; Sedelnikova et al. 2003; Kinner et al. 2008; Dickey et al. 2009). Some of these proteins are involved in all repair mechanisms, while others are specific to certain pathways. A representative of a “universal” repair protein is 53BP1, which plays a role in both non-homologous end joining (NHEJ) and homologous recombination (HR) and appears to be crucial for switching between these mechanisms (Anderson et al. 2001; Bunting et al. 2010; Kakarougkas et al. 2013; Reindl et al. 2015; Bártová et al. 2019). Conversely, a specialized repair protein is RAD51, which participates exclusively in HR, functioning later in this process to search for homologous sequences and form the triplex structures that serve as the substrate for repair (Reindl et al. 2015; Cruz et al. 2018; Schwarz et al. 2019; Wassing and Esashi 2021). Therefore, RAD51 occurs only in late S/G2 cells at DSB sites repaired via HR. The mechanisms by which specific proteins select IRIFs for binding are still unclear, and the decision-making process of cells regarding which repair system to utilize for specific DSBs remains one of fundamental questions of current radiobiology (Shibata et al. 2011; Schipler and Iliakis 2013; Jachimowicz et al. 2019; Scully et al. 2019; Falk and Hausmann 2020b; Frigerio et al. 2023; Mladenov et al. 2023; Van Bueren and Janssen 2024; Vergara et al. 2024; Kumari et al. 2025). Given that radiation-induced DNA damage and subsequent repair occur within the context of the chromatin environment, it is reasonable to hypothesize that this environment plays a significant role in these processes (Falk and Hausmann 2020b).

We have previously demonstrated that the architecture of chromatin significantly influences both the generation and nature of DNA damage and the efficiency and accuracy of repair processes (Fig. 5) (Falk et al. 2007, 2008b, 2010). How chromatin architecture influences the extent and nature of radiation-induced DNA damage has been discussed in previous chapters. Regarding the repair of DSBs, together with others (e.g., (Goodarzi et al. 2010; Goodarzi and Jeggo 2012)), we have found that DSB repair in heterochromatin occurs significantly more slowly than in euchromatin (Falk et al. 2008b, 2014c). In the case of heterochromatin, decondensation must first take place to allow the penetration of specific proteins to the site of the DSB (Falk et al. 2007). However, this decondensation also increases the mobility of heterochromatic DSBs, leading to their protrusion into regions of the cell nucleus with lower chromatin density. In these areas, the interactions between multiple DSBs may occur, potentially resulting in chromosomal translocations. Furthermore, the decondensed heterochromatin must return to its original structural state after the completion of repair. Thus, chromatin architecture evidently influences both the kinetics (Falk et al. 2008b) and accuracy (Falk et al. 2007) of repair, as well as the likelihood of interactions between individual DSBs—specifically, the probability of specific chromosomal translocations forming, which depends on the local chromatin architecture between particular DSBs (Falk et al. 2007, 2010) (summarized in Fig. 5).

Additionally, our preliminary results suggest that chromatin architecture may play a pivotal role in the decision-making process regarding the optimal repair mechanism for each individual DSB site (Falk and Hausmann 2020b). We have revealed that ionizing radiation-induced foci (IRIFs) associated with heterochromatic (H3K9me3) markers exhibit greater mutual similarity in their nanoscale topology compared to euchromatic IRIFs (Bach et al. 2017). Moreover, the results indicate that γH2AX and 53BP1 repair foci (IRIFs) differ in their morphological parameters even at the microscale based on their association with either non-homologous end joining (NHEJ) or homologous recombination (HR) (Falk et al. 2018). Although further research is essential, it is reasonable to hypothesize that the local architecture of chromatin at the site of a DSB may influence the accessibility of specific repair proteins to the DSB lesion and subsequently affect the assembly and development of repair complexes (i.e., IRIF foci).

## Chromatin architecture and the radioresistance of different cell types

It is not surprising that various types of normal cells and different tumor cells respond differently to irradiation—some are highly sensitive, such as lymphocytes (Trowell 1952; Heylmann et al. 2021; Paganetti 2023), while others are highly radioresistant, such as neurons or glioblastoma cells (Shen et al. 2015; Alhaddad et al. 2023). The radiosensitivity or radioresistance of cells depends on numerous factors, including their proliferation rate, sensitivity to apoptosis, DSB repair capacity, etc. (Wu et al. 2023). In tissues, additional phenomena, such as oxygenation levels, can strongly influence radiosensitivity.

The correlation between cell proliferation speed and radiosensitivity (the Bergonié and Tribondeau law) even inspired the initial idea of utilizing IR for tumor elimination (Vogin and Foray 2012). However, the tumor cells frequently demonstrate greater resistance to irradiation than normal cells, despite proliferating faster and having some defects in DNA repair pathways (Pavey et al. 2013) and cell cycle checkpoints (Molinari 2000; Fernando et al. 2021).

This observation suggests that other factors may compensate for these defects, such as tissue hypoxia, increased resistance to apoptosis, overactivation of remaining functional repair pathways, and the ability to tolerate imprecise repair (Wu et al. 2023). In conjunction with previous findings indicating that chromatin structure influences sensitivity to radiation-induced damage (Falk et al. 2008b) and the repair of resulting DNA double-strand breaks (DSBs) (Falk et al. 2007), it is reasonable to hypothesize that chromatin architecture also plays a role in (co)determining cell-type-specific radioresistance.

Using super-resolution microscopy (SMLM), we demonstrated that the formation of repair foci by the DSB repair protein 53BP1 is delayed in U87 glioblastoma cells (compared to normal human skin fibroblasts), though they remain highly radioresistant (Bobkova et al. 2018). Comparing changes in the chromatin architecture of these tumor and normal cells over time following irradiation reveals distinct responses to radiation (Falk et al. 2025a). When quantifying these changes as movements in topological space through principal component analysis (PCA) (Weidner et al. 2023), we observe that while chromatin architecture undergoes closed cycles in normal cells, these “cycles” remain open in tumor cells. Consequently, the chromatin architecture in irradiated tumor cells does not revert to its original state even after DSB repair is complete (Fig. 5, bottom panel) (Falk et al. 2025a).

This persistent alteration may help explain the increased genomic instability observed in tumor cells post-irradiation. Genomic instability is a fundamental characteristic of cancer cells, but both low-LET and high-LET ionizing radiation can induce this phenomenon in normal cells and further exacerbate it in cancer cells (Morgan et al. 1996; Karotki and Baverstock 2012; Yue et al. 2013). The radiation-induced genomic instability manifests as a delayed wave of various instability endpoints, the formation of which is not directly linked to the immediate radiation damage of chromatin. This independence is supported by evidence showing that the nature of immediate post-irradiation DNA damage differs from the delayed damage associated with genomic instability, as evidenced, for instance, by the distinction between chromosome breaks and chromatid breaks. Some researchers, however, suggest that genomic instability arises from complex DNA lesions induced by IR that remain unrepaired (Mavragani et al. 2019). The genomic instability can persist for more than 30 cell generations or passages following irradiation and encompasses a wide range of molecular, cytogenetic, biochemical, morphological, and functional changes. Notable manifestations include increased chromosomal aberrations, gene mutations, clonal heterogeneity, reduced plating efficiency, variable colony sizes, cell fusions, giant cell formation, delayed reproductive cell death, and cell transformation (Morgan et al. 1996; Yue et al. 2013).

Radiation-induced genomic instability can significantly increase cell mutation rates, with factors ranging from ten to ten thousand (Morgan et al. 1996), thereby enhancing the production of radioresistant clones (Morgan et al. 1996). However, the mechanisms underlying radiation-induced genomic instability and its various endpoints remain poorly understood, despite decades of recognition of genomic instability as a significant phenomenon. The factors contributing to this instability include induced telomere instability, altered metabolic processes, epigenetic modifications, or imprinting alterations that lead to reduced DNA repair capacity, impaired DNA replication, and errors in chromosome segregation (Morgan et al. 1996; Karotki and Baverstock 2012).

Importantly, genomic instability is not limited to specific chromosomes or genes; rather, it constitutes a systemic phenomenon that impacts the entire genome. This observation aligns with the persistent pan-nuclear chromatin alterations we have documented in irradiated cancer cells (Falk et al. 2025a, b), and their potential consequences. As outlined in Chapter 6, we propose that the enduring disruption of chromatin architecture (decompaction) in cancer cells may further exacerbate their genomic instability, for example, by facilitating the activation of transposons. The radiation-induced changes in chromatin architecture were more pronounced in radiosensitive cells (Falk et al. 2025a), further supporting the role of chromatin organization in co-determining cellular responses to IR. At the same time, these changes indicate that various endpoints of genomic instability affect radiosensitivity in specific ways. Indeed, significant alterations in chromatin architecture may occur early in carcinogenesis, even in premalignant cells, and may be accompanied by increased radioresistance compared to their normal counterparts (Lukasova et al. 2004, 2005; Cherkezyan et al. 2014; Gruber et al. 2019; Gridina and Fishman 2022; Terekhanova et al. 2023), further highlighting the role of chromatin architecture in determining cell-type-specific radioresistance. However, while both normal and cancer cells experience radiation-induced genomic instability, only cancer cells exhibit persistent pan-nuclear alterations in chromatin architecture, as observed in our nanoscopic studies (Falk et al. 2025a). This suggests that additional mechanisms may be involved in the induction of this intriguing phenomenon.

In cancer cells, the additional factors acquired during the transformation process, as well as those originating from the tumor microenvironment, can significantly influence radiosensitivity by affecting chromatin architecture, in addition to the effects of ionizing radiation. One such factor is hypoxia (Johnson and Barton 2007; Collier et al. 2023). As previously mentioned, hypoxia enhances radioresistance by significantly reducing the production of radiation-induced reactive oxygen species (ROS). Furthermore, hypoxia is known to promote alterations in chromatin architecture (Johnson and Barton 2007; Collier et al. 2023), although the specific effects of hypoxia on chromatin and their implications for radioresistance are still poorly understood. Various studies have reported globally increased histone methylation and deacetylation associated with pan-nuclear chromatin condensation. However, histone acetylation has also been observed under hypoxic conditions (Pouikli et al. 2022), suggesting opposing effects on chromatin structure. In conjunction with epigenetic histone modifications, the formation of cell-type-specific chromatin contacts and the activation of specific genetic loci have been documented (Johnson and Barton 2007; Collier et al. 2023). While chromatin condensation, primarily associated with histone methylation and deacetylation, may enhance the radioresistance of hypoxic cells, the impact of other epigenetic modifications and altered transcription profiles—including those of non-coding RNAs—remains uncertain (Stone et al. 2019). Additionally, the extent to which these chromatin modifications, particularly condensation, relate to hypoxia or hypoxia-induced apoptosis still requires further investigation (Johnson and Barton 2007; Collier et al. 2023).

Figure 5 delineates the key aspects of the relationship between the physical characteristics of ionizing radiation, chromatin architecture, DNA double-strand break (DSB) generation, DSB repair mechanisms, the formation of chromosomal aberrations, post-irradiation genomic instability, and the overall radioresistance of both normal and tumor cells, as discussed throughout this paper. Irradiation elicits both local chromatin responses at the sites of DSBs (top and middle panel) and global reactions (bottom panel), wherein the chromatin network functions as an integrated system.

## Summary

In this article, we outlined a small part of the significant progress in knowledge that radiobiology has achieved over the past 70 years since the establishment of the Institute of Biophysics, Czech Academy of Sciences. From initial experiments at this institute focusing on the simple survival of model organisms and the damage to their hematopoiesis, we have jumped to molecular processes at the level of chromatin with a single molecule resolution. Specifically in this manuscript, we provided a current view on the fundamental relationship between the physical properties of various types of ionizing radiation, chromatin architecture, chromatin damage, the mechanisms of chromatin (DNA) repair, and biological impacts. The presented results prove that different types of radiation damage various chromatin domains in distinct ways.

We have demonstrated that chromatin architecture represents a significant phenomenon in radiobiology, as it influences DNA sensitivity to radiation-induced damage, the mechanism of DNA repair, the formation of chromosomal translocations, and the likelihood of translocations occurring between specific genetic loci. Moreover, it appears that chromatin architecture at the sites of individual DSBs contributes to the decision-making process for selecting the most appropriate repair mechanism in these areas. Furthermore, the overall chromatin network functions as a system that also plays a role in the cellular response to radiation-induced DNA damage, alongside the damaged chromatin domains (summarized in Fig. 5).

Acquiring this knowledge depends on the multiparametric analysis of the microscopic and nanoscale characteristics of DSB repair foci (IRIF), which is not feasible without appropriate software and advanced computational methods. In an era of rapidly advancing AI, it may seem surprising that the detection and analysis of IRIF still pose significant challenges, even for expensive software packages from reputable companies. Therefore, our efforts in developing the DeeFoci software, based on artificial neural networks and deep machine learning, make an important contribution to the analysis of IRIF (Vicar et al. 2021). This software is capable of automatically detecting and performing micro-level analyses of IRIFs composed of γH2AX and various repair proteins, including their mutual colocalization, among other features. Additionally, entirely new mathematical approaches were necessary for the analysis of data from single-molecule localization microscopy (SMLM) at the nanoscale level (Weidner et al. 2023).

It is evident that the chromatin response to irradiation differs among normal and various tumor cells, which may be associated with disparities in genomic instability and cellular radioresistance. Despite significant advancements in understanding the cellular processes triggered by radiation at the molecular level, many fundamental questions in radiobiology remain unanswered, with new inquiries continuously emerging. For instance, the health effects of ionizing radiation at low and very low doses remain uncertain, as multiple opposing processes interact in terms of health risks and benefits.

Following the discovery of bystander and abscopal effects (Morgan 2003; Mancuso et al. 2012; Morgan et al. 2016; Daguenet et al. 2020), it is now clear that the impacts of ionizing radiation are not limited to directly irradiated cells. At higher levels of complexity, we are progressively uncovering, for instance, the increasingly critical role of the immune system, whose response to irradiation is more complex than previously understood. On one hand, we delve into the microcosm and even the nanocosm of molecular processes in cellular responses to irradiation. So, at the same time, we face the expanding complexity and details of knowledge.

Hence, a primary task for radiobiology in the foreseeable future will not only be the advancement of knowledge regarding the biological effects of ionizing radiation across different levels of complexity but also the integration of expanding findings from these various research levels. This will require a distinctly interdisciplinary approach, as exemplified by the European COST MultiChem project, which involved researchers from IBP CAS and whose comprehensive outcomes are summarized in a comprehensive review published in (Solov’yov et al. 2024).

In terms of estimating health risks from radiation exposure for specific individuals (e.g., to select resistant individuals for missions to Mars) and the realm of personalized medicine (e.g., to exclude hypersensitive patients or patients with radioresistant tumors from radiotherapy before treatment), it will be crucial to identify various determinants of radiosensitivity or radioresistance in cells, tissues, and organisms.

An additional important aspect of radiobiological research, whose significance is dramatically increasing today due to advances in cancer radiotherapy, planned lunar and Martian missions, and the current global geopolitical situation, is the development of tumor-specific radiosensitizers and new effective radioprotectors. So, we find ourselves once again at the same point we were 70 years ago, yet several levels higher on the spiral of expanding knowledge, fortified by significantly enhanced and improved experimental capabilities. It would be fascinating to see what knowledge readers will have when revisiting this review in another 70 years. Let us just add, in the context of this article, that certain radioprotectors (e.g., amifostine, (Hofer et al. 2016) and radiosensitizers (e.g., metallic nanoparticles, (Štefanciková et al. 2016; Pagáčová et al. 2019; Dobešová et al. 2022) mediate at least part of their effects by influencing chromatin architecture.

## Data Availability

This paper is a review based on data from recent and older authors' sources, as well as information from other researchers. The recent data are available upon request from the corresponding author, Martin Falk.
